# Assessment of electrical activity in the supra and infra hyoid muscle in individuals with type 2 diabetes mellitus

**DOI:** 10.1186/1758-5996-7-S1-A14

**Published:** 2015-11-11

**Authors:** Tiago Lima Santos, Patricia Maria Mendes Balata, Lucas Albuquerque, Marina Cerqueira Rosdaibida Gomes, Deniele Bezerra Lós, Lucio Vilar, Sílvia Regina Arruda de Moraes

**Affiliations:** 1 Universidade Federal de Pernambuco, Recife, Brazil

## Background

It is believed that muscle changes caused by Diabetes mellitus can impact supra and infra-hyoid muscles, reflecting the phonation process. However, there are no studies evaluating the behavior of the extrinsic muscles of the larynx in phonation activities in this population.

## Objective

To evaluate the electrical activity of supra and infra-hyoid muscles in individuals with type 2 diabetes mellitus.

## Materials and methods

Sample was composed of thirty female adults, aged between 40 and 60 years, distributed in Diabetes group and Control group and they were submitted to a electromyography exam with electromyography MIOTEC® (Rio Grande do Sul, Brazil) connected to a notebook SAMSUMG brand provided the Miotool 200® software, using windowing 32 and gain equal to 2000 for each channel. An electrode reference and three channels connected to active sensors with connection SDS500® by claws were used. The signal analysis was performed with the Miograph 2.0® software. For normalization of the supra-hyoid group (HS) swallowing incomplete maneuver and the infra-hyoid (IH), the tongue retracted technique was used. Then, it was performed the catchment of the muscular electrical activity through the rest moment following by the evaluation during vowel/ε/vocalization and the usual speech. It had been used the t test to calculate the differences between the averages of electrical activity of the groups evaluated.

## Results

There was a reduction in the electrical activity of supra and right and left infra-hyoid muscles during vocalization of the vowel/ε /, normalized by rest moment and reduced electrical activity during normal speech (count 1 to 10) normalized by rest moment only the supra-hyoid muscle in the diabetic group (p <0.05).

## Conclusion

Results suggest that diabetes promotes reduction of muscle electrical activity in phonation activities that are detected when they are normalized by rest moment.

